# Monthly Summary

**Published:** 1899-10

**Authors:** 


### Monthly Summary.

A MOUTHFUL OF TEETH.—To go to bed one night and wake up the next morning with an almost complete extra set of teeth in the mouth is an experience not vouchsafed to many persons. Such a case, however, has been for some time engaging the attention of the officials of one of the London hospitals. The patient is a boy of seventeen years of age, who works in an office in the center of the city. In appearance he is no different from the hundreds of other youths who work about him, but, unlike them, he is an object of peculiar interest to over a hundred doctors and embryo dental surgeons. His story is a peculiar one, and were it not backed up by substantial proof, would be dismissed as widely imaginative.

Some little time ago he began to be affected with acute toothache, but as all his teeth were apparently sound, no local cause could be found by his parents or friends. Being too poor to secure medical attention, he was forced to bear his pain with what fortitude he coulk.

Finally, his gums began to swell on the outer side, and these grew worse until one morning he awoke to find the soreness and pain gone, and that he had an almost duplicate set of teeth growing on the outside of both the top and bottom side of the natural ones. At first this did not interfere with him but later it was found that his speech was beginning to get imperfect. An examination revealed the fact that the new teeth were growing to an abnormal size, while in proportion as they increased, his physical strength decreased.

He was then taken to the hospital, where after careful watching as an out-patient, it was decided to extract a number of the teeth. This was done, but almost as fast as they were taken out new ones grew in other places, until almost the entire roof of the patient's mouth had been taken up by this peculiar ailment, and recently it has been noticed that the growth of further teeth has been stopped. Whether this condition will be permanent is a mooted question time alone will answer, but, meanwhile, the extraction of his teeth has ceased.

The case is remarkable in medical annals, and is set down as a peculiar form of ossification. It is supposed to have a hereditary bearing, the lads' grandfather being described as a peculiarly "double jointed" man of unusual strength. A load of six hundred weight is reputed to be of little weight to him. —Pearson's.

---

PAINLESS EXTIRPATION OF LIVE PULPS WITHOUT CATAPHORESIS.—With the increasing tendency to the removal of all pulps that seem likely to die of themselves within a comparatively short time, there comes a demand for some practical and reliable method by which this operation can be performed without pain and without injuring the important pericemental membrane. Devitalization by means of arsenical compounds has proven to be a very unsafe method, to say the least, permanent injury to the pericementum often resulting. We naturally turn to cocain for a solution of the problem, and applied cataphoretically the great obstacle to its use, the impenetrability of dentine, is overcome. The use of cataphoresis is, however, attended by many difficulties, and it is safe to assume that owing to these difficulties there are comparatively few dentists using the method to-day. Partial devitalization with arsenic and the removal of the greater portion of the pulp with crystals of cocain or a strong solution is usually painless, leaves the pericementum intact and presents so few difficulties as to recommend itself over cataphoresis in ordinary practices. It is often desirable, however, to remove the pulp without the delay necessary in the use of arsenic, and in cases of this kind I have used a solution of cocainhydrochloride in alcohol and ether. This solution either owing to an increased capillarity over the fluid in the dentinal tubuli or some other cause, obtunds thin layers of the dentine so that they may be removed or drilled through, applying the solution as soon as the slightest sensation appears, until the pulp is reached without pain. After gaining access to the pulp a few cocain crystals dissolved in the blood that follows puncture, and carefully pumped into the canal with a broach obtunds the remaining fibres. Either of these methods is extremely simple, safe, and requires little time.—P. M. Williams, D. D. S.—"Dominion Dental Journal."

---

A LABORATORY HINT.—In preparing a full upper or under denture for investment in the vulcanizing flask, and when the set is finally waxed up, take a piece of waxed floss silk and, commencing at the condyle, lay it closely on where the wax terminates on the top, or, if an under set, at the bottom of the rim. Carry the wax thread entirely around the rim and bring the other end of the thread back over the other condyle.

The set can then be invested on the under side of the flask, and the plaster allowed to flow up so as to cover the silk, which is now around the set at the line where the division is to be made between the two parts of the mold.

When the plaster has stiffened just a trifle, place the thumb of the left hand in the center of the set and the fingers on the bottom of the flask and invert it. Then taking the silk by one of the ends, and drawing it firmly but gently taut, pull it away from the set, keeping the silk touching the edge of the flask.

The silk can be drawn in this manner all around the set so as to cut off all surplus plaster very evenly, and so that the excess will drop into the waste-box.

This accomplishes the same work as the knife, and does it much better, doing away with the danger of marring the wax with the knife, and also bringing the plaster more accurately to the top of the set.

The wax around the side of the set can be chalked, which will facilitate the detachment and dropping off of the surplus plaster.—R. Brittain, Cosmos.

---

TEETH MADE OF PAPER.—LATEST THING IN DENTISTRY ARE PAPIER-MACHE MOLARS.—Paper teeth are the latest thing in dentistry. For years some substance has been sought for which could replace the composition commonly employed for making teeth, and a fortune awaited the man who was lucky enough to hit upon the right material. Although paper has some disadvantages, they are all small compared to its many qualifications, and paper teeth are likely to be used exclusivly, at least until a more perfect material is found.

Up to this time, says the New York Press, china has been used almost entirely, but it presents so many disadvantages that dentists always have been on the lookout for some other substance which could replace it. Not only does china not resist the action of saliva and turns back, but china affects the nerves of the jaws. People who wear false teeth often complain of sub-orbital neuralgia, and this is put down by many dentists as being caused by the heat or cold acting on the china or porcelain. Porcelain or mineral composition also is liable to chip or break, and for these reasons have never been satisfactory.

The paper teeth are made of papier-mache, which is submitted to a tremendous pressure until they are as hard as required. Their peculiar composition renders them cheap, and the price of a set of teeth will go down considerably, owing to the new invention. The color of the papier-mache can also be made to vary, which is an important point, as no two sets of teeth are identical in color, some teeth having a strong yellowish cast, while others are bluish white. In order, therefore, to obtain the right tint coloring mater has only to be introduced into the mixture before the tooth is cast, in order to match the other teeth exactly. It is in this particular that china teeth often fail to appear natural, their color differing from the other teeth in the mouth, and showing that the tooth is artificial.

Another novelty with regard to teeth consists in their filling. Dentists no longer use as much gold or platinum as they did formerly—in fact, metal fillings are out of date. Bone or ivory is the substance employed, and both possess the advantage of appearing more natural. Of course, those who already have gold or platinum fillings will not go to the expense and trouble of having them removed, but they have been tabooed by the smart set, and in future nothing so conspicuous will be used. Neither bone nor ivory satisfies the dentists, however, and they are hunting around for some composition which will be both durable and elastic, and yet will match the color of the teeth.

---

REFINING GOLD SCRAP.—To those dentists who wish to refine their gold scrap themselves, the following plan is recommended: The scraps should be dissolved in a small quantity of nitro-muriatic acid—warming hastens the solution—the solution should then be diluted with about three times its volume of water, and nearly neutralized by adding a small quantity of sodium carbonate. The solution should remain slightly acid or the gold will be precipitated; in that case redissolve by adding a few drops of nitro muriatic acid. Filter the solution, washing it through with water, then add slowly while stirring a concentrated solution of ferric sulphate, acidulated with a little sulphuric acid. Set the solution aside for twenty-four hours, so that all the gold is precipitated, then decant the liquor through filter paper to catch any particles of floating gold, wash the precipitate out of the vessel into the filter palter, roll up the paper and fuse with plenty of flux.—Office and Laboratory.

---

TREATMENT OF PORCELAIN FACINGS TO PREVENT FRACTURE.—Porcelain facings in soldering require as much intelligence in handling as does a glass goblet or porcelain dish in washing. A dentist who invests a crown or bridge and as soon as the investment is hard enough to handle places it over the gas stove and puts on a full blast of heat, can feel himself one of the chosen few if he does not always have a broken facing.

The contraction of platinum being more rapid than porcelain in cooling, requires only one consideration in solding, and that is that the flame leaves the point upon the incisive edge where the platinum laps. This insures equitable cooling throughout the tooth, and does away with those minute fractures so often seen upon the incisive portion of the incisors and cuspids when backed with platinum, and so rarely seen on the same teeth backed with gold.

Dry the investment slowly until all traces of moisture are driven off, then increase the heat until the investment is as hot as it is possible for it to get over the gas heater, transfer to an asbestor block, solder, and cover with another asbestos block, and leave until cold.

---

THE RELATION OF DISEASED TEETH TO GENERAL DISEASES.—In a recent work by Oscar Amoeda (Paris, 1898), on the medico-legal aspect of affections of the teeth, there is an excellent chapter on the relation of diseases of the teeth, gums, alveoli, etc., to various general diseases. Infectious diseases, such as la grippe, scarlatina, variola, typhoid fever, erysipelas, etc., provoke dental troubles, which vary in accordance with the patients' age. In teething children trophic or follicular troubles, which, according to their intensity, produce erosions or total loss of the gum Infectious diseases, and particularly the grippe, are frequently accompanied by periostitis and alveolo-dental abscess, and may also cause sinus disease. The alveolo-dental periostitis of diabetes is characterized as an initial sign of that affection by a period of simple deviation. In the second period the teeth loosen with alveolar catarrh. In the third period the teeth fall out. There is sometimes a further state of osseous absorption, which may or may not have been proceded by gangrene of the gum. Besides these lesions the teeth themselves may be affected by caries. This happens often, and begins usually at the last molars. In time, only the roots remaiin to the patient. Arthritic, gouty and rheumatic subjects are predisposed to tartar. Other accidents are alveolar and alveolo-dental periostitis, dental necrosis and fall of the teeth; simple and aphthous gingivitis; alveolar absorption, caries and necrosis of the jaw. Gout is also said to produce wearing away of the teeth. Hereditary syphilis leads to micro-dontism, nauism, amorphism and vulnerability of the teeth. Tuberculosis may attack the gums. In rickets, dental anomalies are not constant; there may be alterations in the enamel, its prisms being disposed sinuously, with large interspaces. The dentine may be remarkable for its large canaliculæ. In locomotor ataxia the teeth often fall out without pain, hemorrhage or suppuration, and irrespective of previous decay. This process begins in the upper jaw, and is generally followed by alveolar resorption. Pregnancy is often accompanied by gingivitis. During menstruation, women, and especially young girls, are subject to attacks of alveolo-dental periostitis. In osteomalacia the teeth rarely are directly involved, but are loosened in the alveoli. In scurvy, the teeth readily loosen and fall out. owing to the state of the gums. In morphinism the ivory suffers chiefly. There is no pain nor periostitis. The course is rapid, the hair often falling at the same time. May be due to central influence or altered saliva. In osteomyelitis of the jaw the pus may gain the alveoli and loosen the teeth, especially the wisdom teeth. The author finishes by alluding to actinomycosis and stomatitis. He fails to mention the effects of persistent nasal obstruction upon the upper teeth.—*Medical Review of Reviews, Feb. 25th, 1899.*

---

CAUSES OF HIGH PALATE.—Grosheintz (*Arch. f. Larygol.*, VIII, No. 3), believes that the high, narrow palate (hypoistaphyly) is usually associated with a generally narrow configuration of the upper part of the face (leptoprosopy). Narrow nasal cavities (leptorrhiny) and narrow orbits are usually associated with high palate. High palate rests, as a rule, upon inborn racial peculiarity of skull formation, and is not influenced by causes operating after birth.—*Medical Review of Reviews, Feb. 25th, 1899.*

ACIDITY OF THE MOUTH DURING SLEEP.—The dentists tell us that an acid condition of the fluids of the mouth plays an important part in the etiology of dental caries; also that the causes of that affection are particularly active during the hours of sleep, when saliva stagnates, so to speak, instead of being subjected to the agitation and renewal incident to the chewing and other movements that to some extent are almost continuous except during sleep. However carefully we may cleanse the teeth and rinse them with antiseptic solutions on going to bed, therefore, we are guarding but temporarily against decay; it gains on us while we are asleep. Possibly those who suffer with insomnia may snatch a crumb of comfort from his reflection, but we fear there is in it no consolation for the mouth-breathers, for the desiccation of the mouth which takes place in them during sleep, while enough to give rise to considerable discomfort on their waking, is quite insufficient to hamper pathogenic bacteria in their work of destruction.—New York Medical Journal, March 18th, 1898.

---

SODIUM SALICYLATE FOR TOOTHACHE.—Dr. Frederick C. Coley, in an article on the medical treatment of toothache in a recent number of the *Practitioner*, states that of all medical remedies for toothache he knows of none which is so successful as salicylate of sodium. He believes it is especially useful in those cases where the pain is started by "taking cold." A dose of 15 grains will usually relieve the pain very promptly and if this is repeated every four hours the inflammation may entirely subside, leaving, of course, a carious tooth to be disposed of according to circumstances. The addition of belladonna is often advantageous. Fifteen grains of sodium salicylate, with fifteen minims of tincture of belladonna, will often procure refreshing sleep instead of a night of agony. It is especially valuable with children, where extraction of teeth is to be avoided, if possible, lest the development of the maxilla should be injured.—*N. Y. Med. Times.*

